# 25 (OH) D3 levels, incidence and recurrence of different clinical forms of benig paroxysmal positional vertigo^☆^

**DOI:** 10.1016/j.bjorl.2017.05.007

**Published:** 2017-06-11

**Authors:** Sinisa Maslovara, Silva Butkovic Soldo, Anamarija Sestak, Katarina Milinkovic, Jasna Rogic-Namacinski, Anamarija Soldo

**Affiliations:** a County General Hospital, Department of Otolaryngology, Vukovar, Croatia; b University of Osijek, Medical Faculty, Department of Otorhinolaryngology and Maxillofacial Surgery, Osijek, Croatia; c Clinical Hospital Center, Department of Neurology, Osijek, Croatia; d University of Osijek, Medical Faculty, Department of Neurology, Osijek, Croatia; e County General Hospital, Department of Laboratory Diagnostic, Vukovar, Croatia

**Keywords:** Benign paroxysmal positional vertigo, Vitamin D3 insufficiency, Recurrence, Clinical forms, Vertigem posicional paroxística benigna, Insuficiência da vitamina D3, Recorrência, Formas clínicas

## Abstract

**Introduction:**

Benign paroxysmal positional vertigo is the most common cause of dizziness in the general population. It is a condition with potential impact of reduced levels of vitamin D on its recurrent attacks.

**Objectives:**

The aim of this study was to measure the serum levels of 25-hydroxyvitamin D3 (25-OH D3) in patients with benign paroxysmal positional vertigo and determine whether there is a difference in the serum levels of vitamin D3 between patients with and without recurrence, as well as between the different clinical forms of benign paroxysmal positional vertigo.

**Methods:**

The study included 40 patients who came to the regular medical examination, diagnosed with posterior canal-benign paroxysmal positional vertigo based on the positive Dix-Hallpike's test. All patients underwent Epley manoeuvre after the diagnosis. Patients were classified according to current guidelines for levels of vitamin D3 in the serum in three groups: the deficiency, insufficiency and adequate level.

**Results:**

The average serum level of 25-OH D3 among respondents was 20.78 ng/mL, indicating a lack or insufficiency of the aforementioned 25-OH D3. According to the levels of 25-OH D3, most patients suffer from deficiency (47.5%). 7 (17.5%) respondents had adequate blood level of 25-OH D3, and 14 (35%) respondents suffer from insufficiency. A significant difference was not found in the serum level of 25-OH D3 between patients with and without benign paroxysmal positional vertigo recurrence. There was a significant difference in the serum levels of 25-OH D3 in comparison to the clinical form of the disease. Lower 25-OH D3 values were found in patients with canalithiasis compared to those with cupulolithiasis.

**Conclusions:**

There were no significant differences in the vitamin D3 serum level in patients with and without recurrence. The study showed a low level of serum vitamin D3 in most patients, indicating the need for supplemental therapy.

## Resumo

**Introdução:**

Vertigem posicional paroxística benigna é a causa mais comum de tonturas na população em geral. É uma condição no qual níveis reduzidos de vitamina D podem ter um potencial impacto para o desenvolvimento de crises recorrentes.

**Objetivos:**

O objetivo desse estudo foi medir os níveis séricos de 25-hidroxivitamina D3 (25-OH D3) em pacientes com vertigem posicional paroxística benigna e determinar se há diferença nos níveis séricos de vitamina D3 entre pacientes com e sem recorrência, bem como entre as diferentes formas clínicas de vertigem posicional paroxística benigna.

**Método:**

O estudo incluiu 40 pacientes submetidos a exame médico regular, diagnosticados com vertigem posicional paroxística benigna de canal posterior baseado no resultado positivo do teste de Dix-Hallpike. Todos os pacientes foram submetidos à manobra de Epley após o diagnóstico. Os pacientes foram classificados de acordo com as diretrizes atuais para os níveis de vitamina D3 sérica em três grupos: deficiência, insuficiência e nível adequado.

**Resultados:**

O nível sérico médio de 25-OH D3 entre os indivíduos avaliados foi de 20,78 ng/mL, indicando falta ou insuficiência desta vitamina. De acordo com os níveis de 25-OH D3, a maioria dos pacientes apresentou deficiência (47,5%). Sete indivíduos (17,5%) entrevistados tinham nível sanguíneo adequado de 25-OH D3 e 14 (35%) apresentavam insuficiência. Não foi encontrada diferença significativa no nível sérico de 25-OH D3 entre pacientes com e sem recidiva de vertigem posicional paroxística benigna. Houve uma diferença significativa nos níveis séricos de 25-OH D3 de acordo com a forma clínica da doença. Baixos níveis de 25-OH D3 foram mais encontrados em pacientes com canalitíase em comparação com aqueles com cupulolitíase.

**Conclusões:**

Não houve diferenças significativas no nível sérico de vitamina D3 em pacientes com e sem recorrência. O estudo mostrou um baixo nível de vitamina D3 sérica na maioria dos pacientes, indicando a necessidade de terapia suplementar.

## Introduction

Benign paroxysmal positional vertigo (BPPV) is the most common cause of dizziness in the general population with a lifetime prevalence of about 10%.^1^ The disease occurs spontaneously, clinically manifests in short seizures, intensive vertigos that are caused by a certain position of the head and are often accompanied by vegetative symptoms. After placing the head in the inciting position, typical vertical-torsional nystagmus can be seen. The illness usually lasts for a few days or weeks and then spontaneously ceases,^2^ but it can also prolong to several months or even exceed and become chronic or recurrent.

Pathophysiological mechanisms of disease development comprise of tearing off crystals of calcium carbonate, otoconia (or otoliths) from the otolithic membrane of the utricle, which then, due to gravity and coinciding with the position of the head, float through endolymph in one of the semicircular canals. Due to the anatomical structure and wide joint front and rear ducts’ parts, otoliths most frequently end up in the rear duct where their mass turns cupular sense, otherwise intended for managing acceleration or deceleration of angular movement, in one sensitive to gravity.^3^ According to the site of accumulation of otoconia, two clinically most prominent forms are canalithiasis and cupulolithiasis, where the former manifests as the accumulation of otoconial debris in the tube itself, while in the latter, the accumulation is directly next to the cupular sense. The cause of the disease is most often idiopathic, in about 50% of cases, and it is associated with degenerative changes associated with the ageing process.^4^ As secondary causes, head injuries lead to the occurrence of BPPV in about 17% of cases,^5^ and viral labyrinthitis (vestibular neuritis) is considered the cause of the BPPV in about 15% of cases.^6^

Epley or Semont manoeuvres or their modifications are mostly used in the treatment of PC-BPPV and their efficiency is reaching almost 100% after two or several procedures.^7^, ^8^, ^9^, ^10^ However, despite the successful repositioning manoeuvre the disease recurs in some patients after a short or longer period, usually taking hold of the same semicircular duct. According to data from the available literature, recurrence happens in 30% of patients after one year,^11^ whereas when longer observation periods are considered relapses are recorded more frequently, in about 50% of cases.^12^, ^13^

In 2003 Vibert D. et al. noted the possible connection between osteoporosis and BPPV.^14^ Additionally, recent research indicated the impact of the vitamin D levels on the BPPV with reduced levels being associated with its occurrence and more frequent recurrence.^15^, ^16^, ^17^, ^18^, ^19^, ^20^, ^21^, ^22^ We know that vitamin D deficiency can cause bone diseases – either as rachitis or osteomalacia. Otoconia, similarly to bones, is a result of the deposition of inorganic calcium carbonate (in the bones it is the calcium phosphate form) on previously formed organic glycoprotein matrix. Although all the details of the otoconia formation are still unknown, it is clear that there is a great similarity between the otoconia and the bone in their matrix structure and the consequent deposition of calcium crystals.^16^ Most experts acknowledge that the 25-OH vitamin D3 plasma/serum concentration is the best indicator of the general supply of the body with vitamin D. While 25-OH D3 form of the vitamin D represents most of the active vitamin D form in plasma/serum, 25-OH D2 form is also present in significant quantities during the replacement therapy with vitamin D2.^23^, ^24^, ^25^ The main objectives of this study were to determine whether there are differences in the serum 25-OH D3 level among respondents suffering from BPPV regarding age, sex, clinical form, and single episode/recurrence of the disease. In addition to measuring the level of 25-OH D3, total serum calcium was determined due to the effect of 25-OH D3 on its level.

## Methods

The study included 40 patients diagnosed with PC-BPPV based on the positive Dix-Hallpike's test coming for their regular medical examination.^26^ All patients underwent Epley manoeuvre after the diagnosis.^27^ The study excluded patients with comorbidities including a confirmed diagnosis of Ménière's disease, vestibular migraine or unilateral or bilateral labyrinth hypofunction, patients taking vitamin D supplements and those with serum calcium abnormalities. Exclusion criteria were applied following patients’ medical history and laboratory findings. Patients were categorised into groups according to the level of vitamin D3. Groups were formed by the new Croatian guidelines for the prevention, detection and treatment of vitamin D deficiency in adults. According to this guideline, the amount that marks the optimal serum level of 25 (OH) D should be above 30 ng/mL. Values between 20 and 30 ng/mL indicate insufficiency, and values equal to or lower than 20 ng/mL indicate a deficiency of 25 (OH) D.^28^

Quantitative analysis of vitamin D and calcium in serum and plasma were performed by standard laboratory method ECLIA (the electrochemiluminescence binding assay). The device used was Cobas e 411 immunoassay analyser (Roche Diagnostics GmbH, Penzberg, Germany). Reference laboratory values for serum vitamin D range from 20 to 50 ng/mL, while the reference values for serum calcium range from 2 to 3 mmoL/L. Serum levels of vitamin D and calcium were measured after the diagnosis of BPPV, and 6 months after that. After examining the specialists’ medical documentation of the patients, other data needed for the research (age, sex, clinical form of the disease and recurrence data) were also gathered. The criteria for recurrence are re-occurrence of symptoms and a positive Dix-Hallpike test after successfully implemented Epley repositioning manoeuvre.

### Ethics

This study was approved by Ethics Committee of the respective institution under an approval protocol number EP-09/2016-4, in accordance with the ethical standards of the institutional and national research committee and consistent with the 1964 Helsinki Declaration and its later amendments, or comparable ethical standards. All of the patients included in the study were adequately informed about the methods and objectives of this study. They have voluntarily accepted to participate in the study. Informed consent was obtained from all individual participants included in the study.

### Statistics

Descriptive statistical methods were used for the frequency distribution of the observed variables. Differences in categorical variables were tested by *χ*^2^ test and, if necessary, by Fisher's exact test. The normality of the distribution of numerical variables was tested by Kolmogorov–Smirnov test. Differences in the normally distributed numerical variables between the two groups were tested by Mann–Whitney *U* test, and according to the diagnoses by Kruskal–Wallis test.^29^, ^30^ All *p*-values are two-sided. The significance level was set at *α* = 0.05. The statistical program R was used in the statistical analysis (www.r-project.org, version 3.2.3.).

## Results

The study included 40 respondents, 29 of whom were women (73%) with mean age of 64. In 19 (47.5%) respondents, the exact clinical form of the disease was determined: 10 (53%) were diagnosed with PC-BPPV (*canalolithiasis*) and 9 (47%) were diagnosed with PC-BPPV (*cupulolithiasis*). Recurrence of the disease was identified in 5 (16%) respondents. The average level of free calcium in the blood was 2.15 mmoL/L and of vitamin D3 20.78 ng/mL (Table 1).

**Table 1:** Characteristics of respondents.

Number (%) of respondents	
*Gender*	
Male	11 (28)
Female	29 (72)

*Recurrence*	
Yes	5 (16)
No	26 (84)

*Diagnosis*	
PC-BPPV (*cupulolthiasis*)	9 (47)
PC-BPPV (*canalolithiasis*)	10 (53)

*Mean (standard deviation)*	
Age (years)	64 (12)
Ca^2+^ (mmoL/L)	2.15 (0.38)
Vitamin D3 (ng/mL)	20.8 (7.87)

Statistically, there was no significant difference between gender groups regarding age, free calcium and vitamin D3 levels (Table 2). Also, significant correlation of recurrence and gender was not found (Table 3). Additionally, no significant differences between recurrence of the disease regarding age, vitamin D3 values and free calcium levels were found (Table 4).

**Table 2:** Mean age, vitamin D3, and free calcium level in blood according to age.

	Mean (standard deviation)		*p*^a^
	Male	Female	
Age (years)	62 (15)	65 (11)	0.842
Vitamin D3 (ng/mL)	21 (6)	21 (8)	0.832
Ca^2+^ (mmoL/L)	2.16 (0.33)	2.14 (0.4)	0.299

a Mann–Whitney *U* test.

**Table 3:** Correlation of recurrence and gender.

	Number of respondents (%)		*p*^a^
	Male	Female	
*Recurrence*			0.583
Yes	2 (25)	3 (13)	
No	6 (75)	20 (87)	

a Fisher's exact test.

**Table 4:** Correlation of mean age, free calcium and vitamin D3 level and recurrence.

Median (interquartile range)			*p*^a^
	According to the incidence of recurrence		
	Recurrence	Without recurrence	
Age (years)	58 (52–74)	65 (57–69)	0.707
Vitamin D3 (ng/mL)	21.9 (14–22)	20.2 (16.8–30.5)	0.485
Ca^2+^ (mmol/L)	1.99 (1.55–2.20)	2.32 (2.16–2.43)	0.068

a Mann–Whitney *U* test.

When analyzing the data according to the diagnosis the following results were obtained: the age of respondents and the level of free calcium did not show significant differences but vitamin D3 level was significantly decreased in PC-BPPV *lat. dex.* (*canalolythiasis*), and significantly increased in PC-BPPV *lat. sin*. (*cupulolithiasis*) (Kruskal–Wallis test, *p* = 0.034) (Table 5).

**Table 5:** Mean and dispersion according to the diagnosis.

	Median (interquartile range)				*p*^a^
	PC-BPPV *lat. dex.* (*canalolithias*)	PC-BPPV *lat. dex.* (*cupulolithiasis*)	PC-BPPV *lat. sin.* (*canalolithiasi*)	PC-BPPV *lat. sin.* (*cupulolithiasi*)	
Age	71 (67–74)	68 (62–69)	66 (61–69.5)	55 (55–62)	0.059
Vitamin D3 (ng/mL)	15 (12.8–18)	24 (22–32)	20.35 (17.75–28)	32 (17–37.8)	**0.034**
Ca^2+^ (mmoL/L)	2.28 (1.7–2.43)	2.32 (2.31–2.38)	1.8 (1.23–2.38)	2.44 (2.37–2.45)	0.500

a Kruskal–Wallis test.

**Figure 1:**
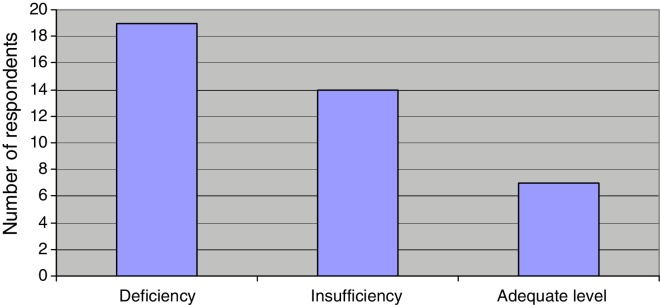
Respondents according to the vitamin D3 level.

According to the levels of vitamin D3, most respondents suffered from its deficiency (47.5%). Adequate blood levels of vitamin D are present in 7 (17.5%) patients while 14 (35%) respondents suffered from vitamin D3 insufficiency (Fig. 1).

Respondents with canalithiasis had significantly lower values of vitamin D3 when compared to cupulolithiasis (Mann–Whitney *U* test, *p* = 0.013), whereas the level of free calcium was similar in both clinical form groups of the disease (Table 6).

**Table 6:** Mean and dispersion according to clinical form of the disease.

Median (interquartile range)			
	According to clinical form of the disease		*p*^a^
	Canalolithiasis	Cupulolithiasis	
Vitamin D3 (ng/mL)	18 (15–20.1)	27 (22–32)	0.013
Ca^2+^ (mmoL/L)	2.27 (1.23–2.39)	2.38 (2.31–2.44)	0.650

a Mann–Whitney *U* test.

In the analyses of the disease clinical form, in cases of canalolithiasis there were significantly more respondents with vitamin D deficiency (6/9 respondents), while in most cases of cupulolithiasis results indicated insufficiency in vitamin D levels (5/10 respondents) (*χ*^2^ test, *p* = 0.036) (Table 7).

**Table 7:** Mean and dispersion according to clinical form of the disease.

Number (%) of respondents according			
Vitamin D3	To clinical form of the disease		*p*^a^
	Canalolithiasis	Cupulolithiasis	
Deficiency	6/9	1	
Insufficiency	2/9	5/10	0.036
Adequate level	1/9	4/10	
Total	9/9	10/10	

a *χ*^2^ test.

## Discussion

The results of this study indicated that the average age of respondents is 64 years, which is consistent with data in the literature. Previous studies suggest that the occurrence of BPPV peaks in the sixth and seventh decade of life.^31^ This study included 40 patients, 27.5% of whom were men and 72.5% women. Although it is known that BPPV occurs twice as often in women,^31^ the difference between women and men in this study can be attributed to the relatively small sample. Also, participation in the study is voluntary and women have responded in higher numbers.

Recent studies have demonstrated the potential impact of reduced levels of vitamin D in the occurrence of BPPV^15^, ^16^, ^17^, ^18^, ^19^, ^20^, ^21^ and more frequent incidence of recurrence in patients with reduced values of the vitamin D.^21^, ^22^ The role of vitamin D is well-known in the regulation of serum calcium and phosphorus, thereby maintaining the proper bone structure. Similarly to the role of vitamin D in the bone metabolism, there are studies showing the role of vitamin D in otolith metabolism as well as positive correlation of reduced vitamin D serum levels and the emergence of BPPV relapse with low vitamin D levels (≤20 ng/mL) presenting a considerable risk factors for BPPV recurrence.^20^, ^21^, ^22^, ^32^ Because of the influence of vitamin D3 in the regulation of serum calcium levels, both the vitamin D3 and the calcium levels were measured in all patients. The average serum calcium for all respondents amounted to 2.15 mmoL/L, which is the lower limit of normal values (reference serum calcium level is 2.14–2.53 mmoL/L). The average serum vitamin D was 20.78 ng/mL, which is slightly less than the average level of vitamin D3 in the study conducted by Büki et al. (23 ng/mL).^15^ There was no significant difference in the level of calcium and vitamin D3 between men and women. Kahr et al. conducted a study in 2016 and found that 93.5% of patients have levels of vitamin D3 less than 20 ng/mL.^33^ Similarly, a large percentage of patients (80%) with vitamin D3 level less than 20 ng/mL was found in a study by Jeong SH et al.^18^ In our study, the largest percentage of respondents (47.5%) recorded vitamin D3 level of less than 20 ng/mL, i.e. deficiency, while 35% recorded insufficiency and only 17% of respondents recorded adequate levels of vitamin D3 (Fig. 1).

There were differences in vitamin D3 levels in various clinical forms of BPPV, as well as in accordance with the affected side, where the highest median vitamin D3 level was in PC-BPPV in patients with cupulolithiasis on the left side, 32 ng/mL, and the lowest in PC-BPPV in patients with right canalithiasis, 15 ng/mL (Table 5). According to this study, patients with canalithiasis have significantly lower serum levels of vitamin D and calcium than patients with cupulolithiasis. A significant difference was found in measured values and the categorization of the vitamin D3 status according to the clinical form of the disease, whereas significant difference was not found in the level of calcium in the same categorization (Table 6, Table 7). According to the clinical form, in canalithiasis, there is a vitamin D deficiency (66.7%), while in cupulolithiasis results indicate vitamin D insufficiency (50%).

Amor-Dorado J.C. et al. found that 36.5% of recurrence occurs within 48 months.^34^ Brandt T. et al. in their retrospective study of 125 patients, 6–17 years after the diagnosis found 50% of recurrence, of which the largest part (80%) occurred within the first year. They also recorded almost twice the incidence of recurrence in patients in the sixth decade of life than in those in the seventh, and much higher rate of recurrence in women (58%) than in men (39%).^13^ In this study, recurrence was, contrary to the findings of those studies,^13^, ^34^ reported in somewhat lower percentage, i.e. in 16.13% of the respondents, for which there was a history of recurrence after the repositioning manoeuvre. Furthermore, a higher number of recurrence was recorded in women, which is in line with the higher number of women with BPPV included in this study (Table 3). The median age of respondents with recurrence was 58 years. Given that the average value of vitamin D3 in patients included in the study was insufficient, it could be stated that there is a positive correlation between the low levels of vitamin D3 and the occurrence of BPPV. Talaat et al. reported the results of their study, where there is a statistically significant difference in the level of vitamin D3 between patients with and without recurrence of BPPV, which was not confirmed in our study.^35^ This fact can be explained by a relatively short period of monitoring patients for recurrence, particularly in those patients who were included in the research last since, as previously shown,^12^, ^13^, ^34^ the number of recurrences increases with time.

## Conclusion

There were no significant differences in serum vitamin D3 level in patients with and without recurrence. The study has demonstrated a low serum vitamin D3 level in most patients, indicating a necessity of a mandatory supplemental therapy for all patients with the reduced 25OHD3. The patients with a clinical canalithiasis form have manifested significantly lower vitamin D3 values than those with cupulolithiasis, which opens up a new perspective on understanding the otolithic metabolism.

## Funding

This research received no specific grant from funding agencies in the public, commercial, or not-for-profit sectors.

## Conflicts of interest

The authors declare no conflicts of interest.
